# From Persisting Diabetes to the Diagnosis of Pancreatic Cancer: A Case Report

**DOI:** 10.7759/cureus.79408

**Published:** 2025-02-21

**Authors:** Fausto Pinto, Marta Roldão, Ana Margarida Ribeiro, João Oliveira, Ines Bento

**Affiliations:** 1 Internal Medicine, Hospital São Francisco Xavier, Lisbon, PRT

**Keywords:** diabetes, malignant pancreatic cancer, oncology, palliative care, weight loss

## Abstract

The diagnosis of new-onset diabetes mellitus (DM) in individuals with a consumptive syndrome warrants a comprehensive and systematic etiological investigation.

This article presents the case of a 45-year-old male, smoker, with a history of a treated C hepatitis virus, diagnosed with type 2 diabetes, accompanied by complaints of fatigue, anorexia, and unintentional weight loss. Despite an initial workup by the general practitioner, including colonoscopy, endoscopy, and abdominal-pelvic CT scan, no neoplastic etiology was identified. A few months later, due to worsening pain and continued weight loss, along with multiple visits to the emergency room, he was referred for an internal medicine consultation for further evaluation. During this investigation, stage IV pancreatic cancer was diagnosed, with multiple organ involvement, and the patient was indicated for best supportive care. The patient passed away four months after the diagnosis.

Through this clinical case, the authors aim to highlight that in cases of newly diagnosed diabetes mellitus with poor control despite therapy and negative imaging results, and associated risk factors, such as tobacco and virus C hepatitis, continuous etiological investigation is crucial, particularly when there is a failure to achieve metabolic control despite appropriate pharmacological treatment and diet.

## Introduction

The diagnosis of new-onset diabetes mellitus (DM) in individuals with a consumptive syndrome necessitates a thorough and systematic etiological investigation.

The ability to detect pancreatic cancer (PC) at a stage where it is potentially curable is contingent on early identification and the capacity to identify high-risk populations prior to the onset of clinical symptoms. However, the definition of high-risk cohorts remains complex, and the development of optimal screening methodologies continues to be a subject of ongoing research [1]. Advancing age is the most prominent risk factor, with incidence peaking at 65-69 years in males and 75-79 years in females [2].

A significant proportion (>80%) of PC cases are attributed to sporadic somatic mutations, whereas only a minority arise from inherited deleterious germline mutations [1]. Familial PC, defined by the occurrence of at least 2 first-degree relatives diagnosed with the disease, accounts for approximately 4%-10% of all cases [3].

In the context of sporadic cases, the primary risk factors include tobacco use, Helicobacter pylori infection, and dietary habits such as high consumption of red meat, excessive alcohol intake, low intake of fruits and vegetables, obesity, and type 2 diabetes mellitus [2-5]. Chronic pancreatitis, regardless of its underlying etiology, whether alcohol abuse, smoking, or genetic mutations, constitutes a well-established risk factor. It is noteworthy that several risk factors associated with PC are modifiable, presenting a substantial but underutilized opportunity for primary prevention [3].

Approximately 75% of PC cases originate in the pancreatic head, 17%-26% in the body and tail, and the remaining 5%-8% are multifocal [6-7]. Tumors located in the body and tail are typically diagnosed at more advanced stages compared to those in the head, given that tumors in the pancreatic head often present with symptoms resulting from obstruction of the common bile duct and/or pancreatic duct. The most frequently observed clinical manifestations of PC include jaundice (predominantly in tumors of the head), abdominal pain, unexplained weight loss, steatorrhea, and the onset or exacerbation of pre-existing diabetes mellitus.

Computed tomography (CT) remains the imaging modality of choice for both the diagnosis and staging of PC, with comprehensive imaging protocols encompassing the chest, abdomen, and pelvis. In instances of obstructive jaundice attributable to a tumor in the pancreatic head, bile duct dilation serves as a crucial radiological marker for tumor delineation [3].

PCs may originate from both the exocrine and endocrine components of the pancreas; however, approximately 95% arise within the exocrine compartment, primarily from ductal epithelium, acinar cells, or stromal tissue. Notably, only 2% of exocrine pancreatic tumors are classified as benign. The predominant histological subtype is pancreatic ductal adenocarcinoma (PDAC), which accounts for approximately 80% of all PC cases [8-10].

Early-onset cancer (EOC) is usually defined as patients with cancer before the age of 50 years. On one hand, EOCs generally show more malignant and aggressive biological behaviors in general, leading to a worse prognosis. Furthermore, young patients with EOCs are more likely to develop specific germline mutations and adverse genetic abnormalities. Early-onset pancreatic cancer (EOPC) patients appeared to receive more treatment (including surgery, radiation, and chemotherapy), have a more advanced stage, and are more male, smokers, and alcohol users. EOPC was located more frequently in the head of the pancreas. Additionally, EOPC had more family history of pancreatic neoplasia and pain symptoms [11].

In the advanced stages of the disease, several prognostic factors have been identified through landmark phase III clinical trials. These include poor performance status (ECOG PS 2), age >65 years, hypoalbuminemia (<35 g/L), presence of synchronous metastases, hepatic involvement, an increased number of metastatic sites, and elevated serum levels of CA 19-9, all of which are associated with a diminished prognosis and reduced survival rates [12-13].

This general background sets the stage for our case report and illustrates the diagnostic journey from persisting diabetes to the diagnosis of pancreatic cancer, highlighting the need for continuous etiological investigation.

New-onset diabetes (NOD) is a high-risk indicator for pancreatic cancer, although pancreatic cancer itself accounts for less than 1% of NOD cases. However, approximately 80% of pancreatic cancer patients are diagnosed with either hyperglycemia or diabetes. Key factors associated with NOD in the context of pancreatic cancer include age, a family history of pancreatic cancer, previous episodes of pancreatitis or cholecystitis, weight loss, and a rapid increase in blood glucose levels or the need for insulin [14].

## Case presentation

A 45-year-old male of Caucasian descent, with a personal history of a work-related accident (appliance mover) in August 2021, resulting in multiple tendon ruptures in the right upper limb, complicated by complex regional pain syndrome. He has a history of treated hepatitis C virus infection and is an active smoker (31 pack-years).

In November 2023, the patient was diagnosed by his family doctor with type 2 diabetes mellitus (HbA1c - 6.6%) and was prescribed dulaglutide 1.5 mg/mL. He also presented with complaints of fatigue, anorexia, unintentional weight loss (30 kg over the course of 10 months), and incapacitating dorsolumbar pain since October 2023.

Given these symptoms, upper gastrointestinal endoscopy, colonoscopy, and abdominal-pelvic computed tomography (CT) were requested in March 2024 to investigate the possibility of an underlying neoplastic etiology. The results from these examinations did not reveal any significant findings.

Due to the exacerbation of anorexia and poorly controlled chronic pain syndrome, the patient repeatedly sought care at the emergency department and was subsequently referred to the internal medicine clinic for further evaluation.

**Figure 1:**
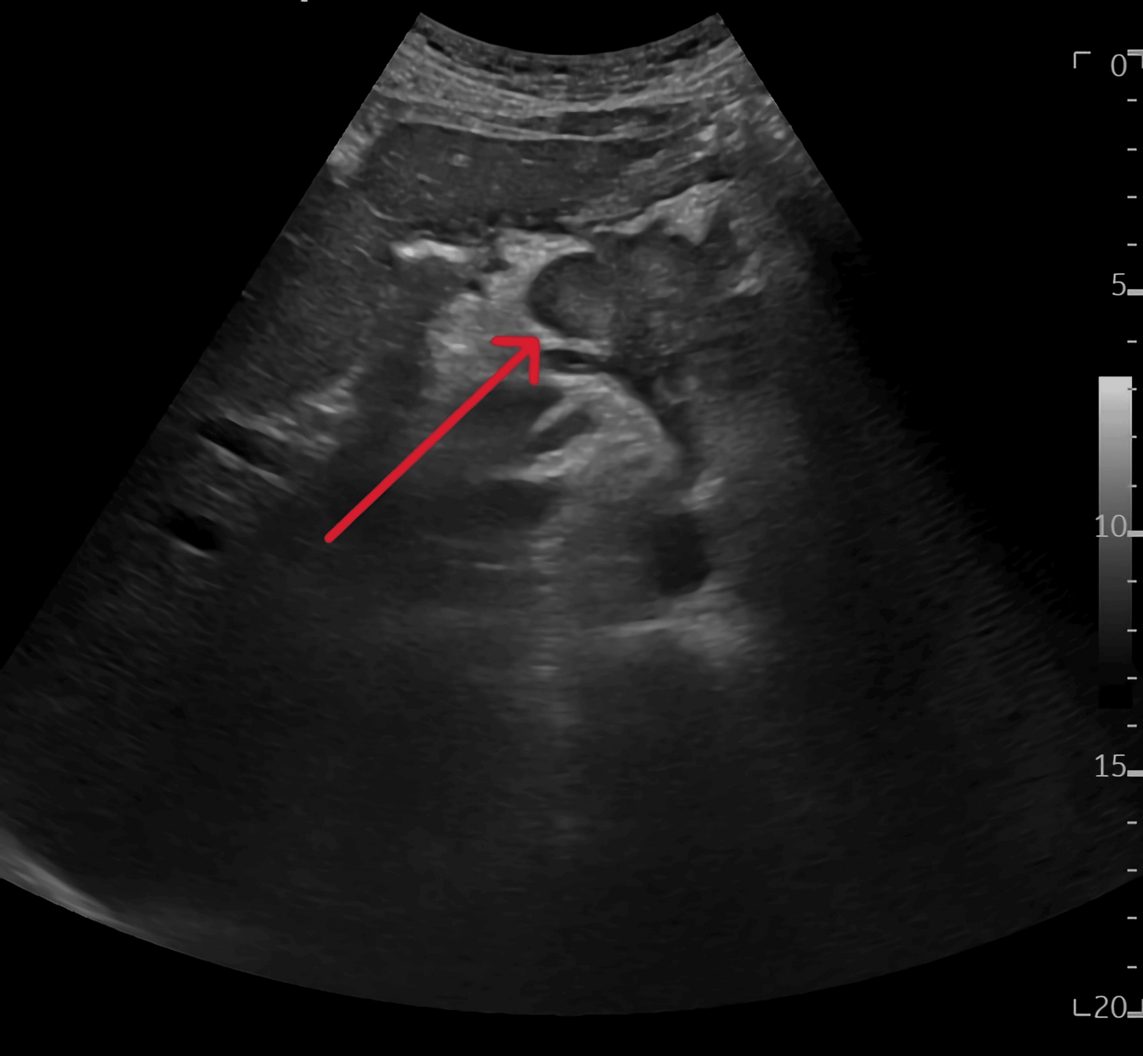
Abdominal ultrasound Solid mass with irregular contours and heterogeneous characteristics

Upon evaluation, analgesic therapy was adjusted, and laboratory tests were requested. The results highlighted isolated thrombocytopenia (76,000 platelets; a condition already noted in prior analyses from 2011), an increased HbA1c of 7.1% (indicating worsening of diabetes control), and no evidence of renal dysfunction. Chronic thrombocytopenic purpura was suspected, and an abdominal ultrasound was ordered in July 2024 to rule out hepatosplenomegaly. The ultrasound revealed "a solid mass with irregular contours and heterogeneous characteristics, measuring 82 mm, located anterior to the bifurcation of the celiac trunk, with splenomegaly appearing homogeneous, with a craniocaudal diameter of the spleen measuring 13.7 cm and a transverse diameter of 14 cm (Figure 1).

**Figure 2:**
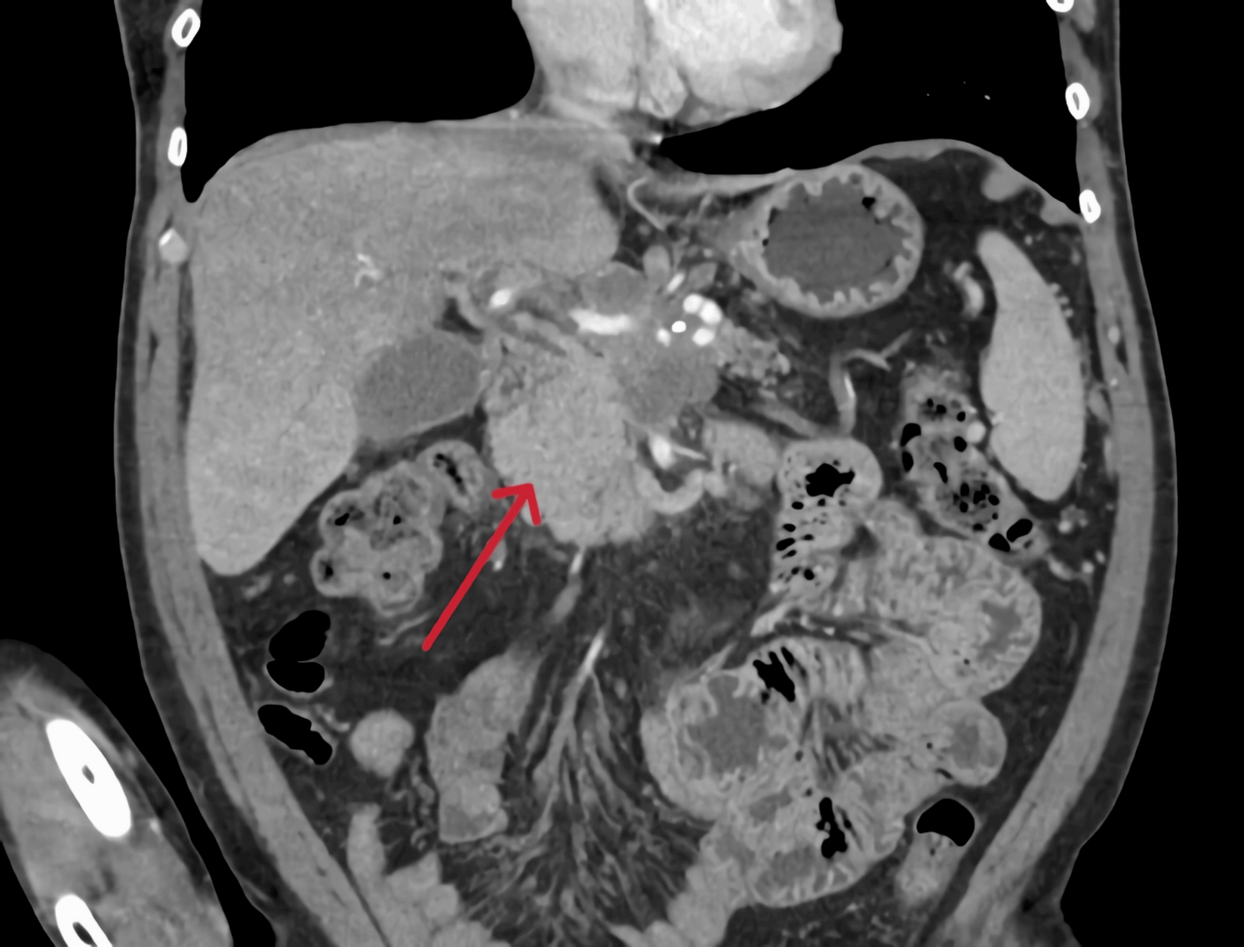
Abdominal-pelvic CT scan Hypodense mass centered in the body of the pancreas, highly suggestive of a locally advanced primary malignant pancreatic tumor

Considering the suspicion of a potential pancreatic neoplasm, an additional abdominal-pelvic CT (Figure 2) was performed in August 2024, which revealed “a hypodense mass centered in the body of the pancreas, highly suggestive of a locally advanced primary malignant pancreatic tumor. There was also the presence of celiac and lateroaortic adenopathy, along with osteoblastic metastasis in the sacrum, both iliac bones, left femur, and lumbar vertebral bodies.

A biopsy was performed using endoscopic ultrasound, confirming the diagnosis of a stage IV pancreatic neuroendocrine tumor, with hepatic, bone, lymph node, and peritoneal carcinomatosis involvement.

The patient was referred to Oncology, Palliative Medicine, and Endocrinology for therapeutic decision-making and continuation of care. After a multidisciplinary discussion, the decision was made to initiate the best supportive care, given the systemic involvement of the tumor. The patient unfortunately passed away approximately four months following diagnosis while hospitalized in Palliative Medicine.

## Discussion

The clinical case presented exemplifies the inherent complexities in medical practice. The patient, exhibiting a clinical presentation (weight loss, newly diagnosed diabetes, fatigue, and anorexia) indicative of a consumptive syndrome, likely secondary to pancreatic neoplasia, underwent an initial diagnostic workup, which, however, did not reveal any significant pathological findings. Given the progressive worsening of symptoms and the strong clinical suspicion associated with risk factors for pancreatic cancer (smoking and past hepatitis C virus), a subsequent imaging study was conducted, which ultimately validated the initial diagnostic hypothesis.

Clinical cases of this nature, involving relatively young adults (45 years of age) diagnosed with malignancies that carry a poor prognosis, pose significant challenges not only from a therapeutic standpoint due to the invasive nature of the tumor and the limited therapeutic options available but also in terms of the communication of the diagnosis to the patient and their family. Confronting a diagnosis associated with a poor prognosis, as in this case, entails considerable emotional and psychological burden.

This case underscores the multifaceted nature of clinical decision-making and reinforces the principle that, in the practice of medicine, the diagnostic process is iterative and dynamic. Despite negative results from initial complementary diagnostic tests, further investigation should not be discontinued when clinical suspicion remains high, as it is critical to pursue the correct etiological diagnosis.

## Conclusions

In conclusion, this clinical case seeks to highlight the potential association between the initial diagnosis of diabetes mellitus in a young patient and the concomitant presence of signs and symptoms indicative of a consumptive syndrome. It is imperative, notwithstanding the results obtained from various complementary diagnostic examinations, to persist in the search for an underlying etiology when the findings fail to align with the primary clinical suspicions.

When suspecting a pancreatic neoplasm, it is essential to consider the most significant risk factors and recognize that, although most cases occur in older individuals, early-onset pancreatic cancer (before 50 years old) is also a possibility. Early detection is critical for improving clinical outcomes.
